# 1,1′-Bis(1-acetyl-5-methyl-1*H*-pyrazol-3-yl)ferrocene

**DOI:** 10.1107/S1600536811041924

**Published:** 2011-10-12

**Authors:** Bei-Bei Zhu, Yao-Cheng Shi, Wen-Bin Shen, Qian-Kun Li

**Affiliations:** a Department of Chemical Engineering, Nantong Vocational College, Nantong 226007, People’s Republic of China; b College of Chemistry and Chemical Engineering, Yangzhou University, Yangzhou 225002, People’s Republic of China; c Analytical Center, China Pharmaceutical University, Nanjin 210009, People’s Republic of China; d Hubei Research Institue of Geophysics Survey and Design, Wuhan 430056, People’s Republic of China

## Abstract

The title compound, [Fe(C_11_H_11_N_2_O)_2_], crystallizes with two independent mol­ecules in the asymmetric unit which have have different conformations. In one mol­ecule, the two ferrocene cyclo­penta­dienyl rings are fully eclipsed and the two pyrazole rings are *syn* to each other; in the other, the two cyclo­penta­dienyl rings are synclinal and the pyrazole rings are *anti*. In both mol­ecules, the acetyl group attached to the pyrazole ring is oriented away from the iron–cyclo­penta­dienyl group of ferrocene.

## Related literature

For background to pyrazole compounds in coordination chemistry, supra­molecular chemistry and organometallic chemistry, see: Chakrabarty *et al.* (2004 ▶); Miranda *et al.* (2005 ▶); Esquius *et al.* (2001 ▶). For related structures, see: Shi *et al.* (2005 ▶, 2006*a* ▶,*b* ▶).
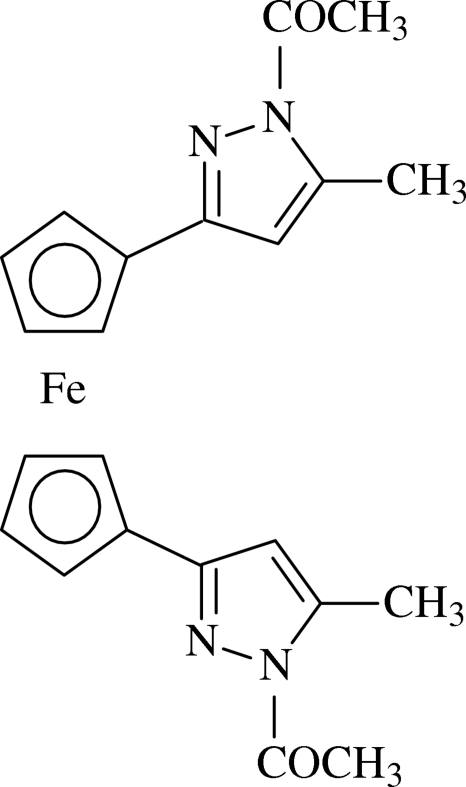


## Experimental

### 

#### Crystal data

[Fe(C_11_H_11_N_2_O)_2_]
*M* *_r_* = 430.29
Monoclinic, 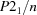

*a* = 10.9021 (19) Å
*b* = 12.7992 (16) Å
*c* = 27.9421 (14) Å
β = 90.492 (16)°
*V* = 3898.8 (9) Å^3^
*Z* = 8
Mo *K*α radiation
μ = 0.80 mm^−1^
*T* = 295 K
0.22 × 0.18 × 0.11 mm

#### Data collection

Enraf–Nonius CAD-4 diffractometer
Absorption correction: ψ scan (North *et al.*, 1968 ▶) *T*_min_ = 0.832, *T*_max_ = 0.907
8056 measured reflections
7639 independent reflections
4199 reflections with *I* > 2σ(*I*)
*R*_int_ = 0.032
3 standard reflections every 200 reflections  intensity decay: none

#### Refinement

*R*[*F* ^2^ > 2σ(*F* ^2^)] = 0.077
*wR*(*F* ^2^) = 0.160
*S* = 1.07
7639 reflections
529 parameters
H-atom parameters constrained
Δρ_max_ = 1.31 e Å^−3^
Δρ_min_ = −0.34 e Å^−3^

### 

Data collection: *CAD-4 Software* (Enraf–Nonius, 1989 ▶); cell refinement: *CAD-4 Software*; data reduction: *XCAD4* (Harms & Wocadlo, 1995 ▶); program(s) used to solve structure: *SIR2004* (Burla *et al.*, 2005 ▶); program(s) used to refine structure: *SHELXTL* (Sheldrick, 2008 ▶); molecular graphics: *PLATON* (Spek, 2009) ▶ and *WinGX* (Farrugia, 1999 ▶); software used to prepare material for publication: *publCIF* (Westrip, 2010 ▶).

## Supplementary Material

Crystal structure: contains datablock(s) I, global. DOI: 10.1107/S1600536811041924/ng5240sup1.cif

Structure factors: contains datablock(s) I. DOI: 10.1107/S1600536811041924/ng5240Isup2.hkl

Additional supplementary materials:  crystallographic information; 3D view; checkCIF report

## Figures and Tables

**Fig. 1.**
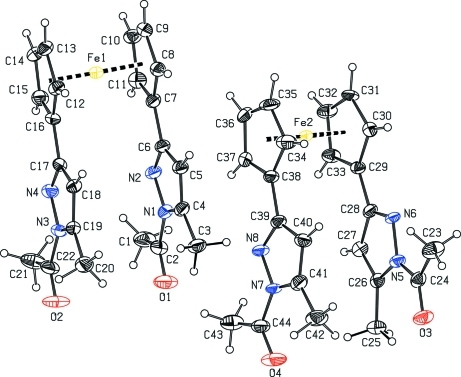
Molecular structure of (I), showing two independent molecules and the 30% probability displacement ellipsoids.

## supplementary crystallographic information

### Comment

The title compound, (I), synthesized from the acetylation of 1, 1'-bis(5-methyl-1*H*-pyrazol-1-yl)ferrocene in the presence of Et_3_N, crystallizes with two independent molecules (Fig. 1) in the space group P 2_1_/n, and the two molecules are rotamers for each other and have different conformations. For each type of molecules (types a and b), two acetyl groups attached to two pyrazole rings are oriented away from the iron-cyclopentadienyl group of ferrocene. However, two pyrazole rings in Ia are *cis* whereas both are *trans* in Ib. Furthermore, the two cyclopentadienyl rings of ferrocene in type Ia are fully eclipsed because the torsion angle C7—C1g—C2g—C16 in Ia (where C1g and C2g are the cycopentadiene ring centroids) is 0.1 (5)° while they are synclinal because the value in type Ib is 7.3 (5)°. The cyclopentadienyl ring and the corresponding pyrazole ring form dihedral angles of 5.4 (3) and 7.7 (3)° for Ia and 3.9 (3) and 3.9 (4)° for Ib. The pyrazole ring and the corresponding acetyl group make dihedral angles of 6.5 (3) and 3.7 (8)° for Ia and 4.3 (7) and 4.5 (8)° for Ib.

As with the reported pyrazole compounds, the bond lengths of each pyrazole ring in (I) indicate electron delocalization (Shi *et al.*, 2005, 2006*a*, 2006*b*). In addition, intermolecular C—H···O hydrogen bonds for each of Ia and Ib are present in the crystalline state (Table 1).

### Experimental

A mixture of 80% hydrated hydrazine (28 ml) and Fe(C_5_H_4_COCH_2_COCH_3_)_2_ (5.313 g, 15 mmol) was stirred for 24 h at ambient temperature under N_2_. The orange-yellow solid was collected, washed with water and air-dried to give 1, 1'-bis(5–methyl–1*H*–pyrazol–1–yl)ferrocene (4.122 g, yield, 79.4%; d.p., 497.75 K).

To a mixture of 1, 1'-bis(5–methyl–1*H*–pyrazol–1–yl)ferrocene (1.385 g, 4 mmol) and Et_3_N (3.036 g, 30 mmol) in 10 ml of THF acetyl chloride (2 ml, 28 mmol) in 5 ml of THF was added dropwise and stirred for 1 h at ambient temperature under N_2_. The resulting filtrate was stripped off solvent and purified by chromatography on silica gel with dichloromethane as an eluant to afford an orange-red solid (0.652 g, yield, 37.9%; m.p., 432.35–433.25 K). Analysis calculated for C_22_H_22_FeN_4_O_2_: C 61.41, H 5.15, N 13.02%; found: C 61.38, H 5.23, N 13.29%. IR (KBr): 3103 (*w*, C—H), 1722 (*vs*, C═O), 1580 (*m*, C═N) cm^-1^. UV (nm, λ_max_, ε(× 10^4^), in DMF): 263.00 (2.86, *B* band), 337.00 (0.49, *K* band), 454.00 (0.07, *d*-*d* band). ^1^H NMR (500 MHz, CDCl_3_, δ, p.p.m.): 5.965 (s, 2H, 2CH), 4.692, 4.347 (s, 4H, s, 4H, (^2^H, ^5^H) and (^3^H, ^4^H) of two C_5_H_4_ rings), 2.658 (s, 6H, 2COCH_3_), 2.519 (s, 6H, 2CH_3_).

### Refinement

All H atoms bonded to parent atoms were placed at geometrically idealized positions and then treated as riding atoms, with C—H distances of 0.93 Å (aromatic), and 0.96 Å (CH_3_) and with *U*_iso_(H) = 1.2*U*_eq_(C), or 1.5*U*_eq_(C) for methyl groups.

The H atoms of C3 and C20 methyl groups were modelled as six equally spaced half-H atoms.

The final difference Fourier map had peak in the vicinity of the Fe atom.

### Crystal data

**Table:**

[Fe(C_11_H_11_N_2_O)_2_]	*F*(000) = 1792
*M**_r_* = 430.29	*D*_x_ = 1.466 Mg m^−^^3^
Monoclinic, *P*2_1_/*n*	Mo *K*α radiation, λ = 0.71073 Å
Hall symbol: -P 2yn	Cell parameters from 25 reflections
*a* = 10.9021 (19) Å	θ = 9–15°
*b* = 12.7992 (16) Å	µ = 0.80 mm^−^^1^
*c* = 27.9421 (14) Å	*T* = 295 K
β = 90.492 (16)°	Prism, red
*V* = 3898.8 (9) Å^3^	0.22 × 0.18 × 0.11 mm
*Z* = 8	

### Data collection

**Table:**

Enraf–Nonius CAD-4 diffractometer	4199 reflections with *I* > 2σ(*I*)
Radiation source: fine-focus sealed tube	*R*_int_ = 0.032
graphite	θ_max_ = 26.0°, θ_min_ = 1.5°
ω/2θ scans	*h* = 0→13
Absorption correction: ψ scan (North *et al.*, 1968)	*k* = 0→15
*T*_min_ = 0.832, *T*_max_ = 0.907	*l* = −34→34
8056 measured reflections	3 standard reflections every 200 reflections
7639 independent reflections	intensity decay: none

### Refinement

**Table:**

Refinement on *F*^2^	Primary atom site location: structure-invariant direct methods
Least-squares matrix: full	Secondary atom site location: difference Fourier map
*R*[*F*^2^ > 2σ(*F*^2^)] = 0.077	Hydrogen site location: inferred from neighbouring sites
*wR*(*F*^2^) = 0.160	H-atom parameters constrained
*S* = 1.07	*w* = 1/[σ^2^(*F*_o_^2^) + (0.0355*P*)^2^ + 7.7275*P*] where *P* = (*F*_o_^2^ + 2*F*_c_^2^)/3
7639 reflections	(Δ/σ)_max_ = 0.001
529 parameters	Δρ_max_ = 1.31 e Å^−^^3^
0 restraints	Δρ_min_ = −0.34 e Å^−^^3^

### Special details

**Table:**

Geometry. All e.s.d.'s (except the e.s.d. in the dihedral angle between two l.s. planes) are estimated using the full covariance matrix. The cell e.s.d.'s are taken into account individually in the estimation of e.s.d.'s in distances, angles and torsion angles; correlations between e.s.d.'s in cell parameters are only used when they are defined by crystal symmetry. An approximate (isotropic) treatment of cell e.s.d.'s is used for estimating e.s.d.'s involving l.s. planes.
Refinement. Refinement of *F*^2^ against ALL reflections. The weighted *R*-factor *wR* and goodness of fit *S* are based on *F*^2^, conventional *R*-factors *R* are based on *F*, with *F* set to zero for negative *F*^2^. The threshold expression of *F*^2^ > σ(*F*^2^) is used only for calculating *R*-factors(gt) *etc*. and is not relevant to the choice of reflections for refinement. *R*-factors based on *F*^2^ are statistically about twice as large as those based on *F*, and *R*- factors based on ALL data will be even larger.

### Fractional atomic coordinates and isotropic or equivalent isotropic displacement parameters (Å^2^)

**Table:**

	*x*	*y*	*z*	*U*_iso_*/*U*_eq_	Occ. (<1)
C1	0.8011 (6)	0.9723 (5)	0.5909 (3)	0.065 (2)	
H1A	0.8593	1.0224	0.5793	0.097*	
H1B	0.7794	0.9895	0.6232	0.097*	
H1C	0.7289	0.9737	0.5710	0.097*	
C2	0.8563 (6)	0.8661 (6)	0.5897 (2)	0.0541 (19)	
C3	0.9242 (5)	0.6313 (5)	0.5988 (2)	0.0502 (18)	
H3A	0.9838	0.6865	0.5975	0.075*	0.50
H3B	0.9353	0.5851	0.5721	0.075*	0.50
H3C	0.9346	0.5931	0.6281	0.075*	0.50
H3D	0.9187	0.5566	0.6010	0.075*	0.50
H3E	0.9672	0.6580	0.6263	0.075*	0.50
H3F	0.9678	0.6500	0.5704	0.075*	0.50
C4	0.7984 (5)	0.6769 (5)	0.5968 (2)	0.0397 (16)	
C5	0.6872 (5)	0.6302 (5)	0.5976 (2)	0.0387 (15)	
H5	0.6720	0.5589	0.6002	0.046*	
C6	0.5983 (5)	0.7103 (5)	0.5939 (2)	0.0373 (15)	
C7	0.4634 (5)	0.7007 (5)	0.5925 (2)	0.0383 (14)	
C8	0.3962 (5)	0.6047 (5)	0.5905 (2)	0.0385 (15)	
H8	0.4299	0.5379	0.5908	0.046*	
C9	0.2693 (6)	0.6291 (5)	0.5881 (2)	0.0431 (16)	
H9	0.2052	0.5813	0.5862	0.052*	
C10	0.2576 (6)	0.7397 (5)	0.5891 (2)	0.0461 (18)	
H10	0.1846	0.7772	0.5884	0.055*	
C11	0.3773 (6)	0.7823 (5)	0.5911 (2)	0.0452 (17)	
H11	0.3960	0.8532	0.5915	0.054*	
C12	0.4054 (5)	0.6172 (5)	0.4699 (2)	0.0394 (15)	
H12	0.4399	0.5508	0.4684	0.047*	
C13	0.2787 (6)	0.6403 (6)	0.4697 (2)	0.0451 (17)	
H13	0.2153	0.5918	0.4677	0.054*	
C14	0.2649 (6)	0.7509 (6)	0.4729 (2)	0.0513 (19)	
H14	0.1914	0.7877	0.4734	0.062*	
C15	0.3853 (6)	0.7943 (5)	0.4752 (2)	0.0469 (16)	
H15	0.4040	0.8649	0.4778	0.056*	
C16	0.4709 (5)	0.7125 (5)	0.4728 (2)	0.0383 (15)	
C17	0.6063 (5)	0.7234 (5)	0.4717 (2)	0.0351 (15)	
C18	0.6946 (5)	0.6416 (5)	0.4709 (2)	0.0353 (14)	
H18	0.6790	0.5702	0.4726	0.042*	
C19	0.8060 (5)	0.6879 (5)	0.4672 (2)	0.0393 (15)	
C20	0.9288 (5)	0.6398 (5)	0.4641 (2)	0.0546 (19)	
H20A	0.9592	0.6472	0.4322	0.082*	
H20B	0.9234	0.5669	0.4721	0.082*	
H20C	0.9838	0.6739	0.4862	0.082*	
C21	0.8154 (6)	0.9840 (5)	0.4578 (3)	0.068 (2)	
H21A	0.8807	1.0334	0.4623	0.102*	
H21B	0.7563	0.9922	0.4827	0.102*	
H21C	0.7766	0.9959	0.4273	0.102*	
C22	0.8660 (6)	0.8763 (6)	0.4592 (2)	0.0537 (19)	
C23	1.0519 (6)	0.5653 (5)	0.8634 (3)	0.063 (2)	
H23A	1.1143	0.5126	0.8618	0.095*	
H23B	0.9902	0.5518	0.8394	0.095*	
H23C	1.0152	0.5642	0.8945	0.095*	
C24	1.1073 (6)	0.6692 (5)	0.8548 (2)	0.0426 (16)	
C25	1.1744 (5)	0.8998 (5)	0.8297 (2)	0.0472 (17)	
H25A	1.2346	0.8470	0.8365	0.071*	0.50
H25B	1.1847	0.9569	0.8516	0.071*	0.50
H25C	1.1845	0.9245	0.7975	0.071*	0.50
H25D	1.1679	0.9719	0.8206	0.071*	0.50
H25E	1.2178	0.8620	0.8054	0.071*	0.50
H25F	1.2180	0.8944	0.8595	0.071*	0.50
C26	1.0483 (5)	0.8544 (5)	0.8350 (2)	0.0358 (14)	
C27	0.9378 (5)	0.9004 (5)	0.8290 (2)	0.0383 (15)	
H27	0.9229	0.9696	0.8206	0.046*	
C28	0.8491 (5)	0.8219 (5)	0.8380 (2)	0.0371 (15)	
C29	0.7148 (5)	0.8317 (4)	0.8354 (2)	0.0324 (14)	
C30	0.6269 (6)	0.7532 (5)	0.8464 (2)	0.0414 (16)	
H30	0.6441	0.6860	0.8573	0.050*	
C31	0.5087 (5)	0.7954 (6)	0.8378 (2)	0.0441 (16)	
H31	0.4349	0.7600	0.8416	0.053*	
C32	0.5208 (5)	0.8981 (5)	0.8227 (2)	0.0506 (18)	
H32	0.4569	0.9437	0.8154	0.061*	
C33	0.6473 (5)	0.9213 (5)	0.8205 (2)	0.0447 (16)	
H33	0.6809	0.9846	0.8108	0.054*	
C34	0.6473 (6)	0.6749 (6)	0.7340 (2)	0.058 (2)	
H34	0.6752	0.6101	0.7445	0.069*	
C35	0.5229 (6)	0.7049 (8)	0.7279 (3)	0.067 (2)	
H35	0.4553	0.6626	0.7337	0.080*	
C36	0.5186 (6)	0.8063 (8)	0.7119 (2)	0.071 (3)	
H36	0.4475	0.8437	0.7049	0.085*	
C37	0.6403 (6)	0.8447 (7)	0.7078 (2)	0.061 (2)	
H37	0.6631	0.9114	0.6982	0.073*	
C38	0.7212 (6)	0.7613 (6)	0.7214 (2)	0.0472 (19)	
C39	0.8554 (6)	0.7657 (6)	0.7200 (2)	0.0454 (18)	
C40	0.9398 (5)	0.6853 (5)	0.7321 (2)	0.0441 (16)	
H40	0.9217	0.6190	0.7437	0.053*	
C41	1.0522 (6)	0.7254 (6)	0.7234 (2)	0.0482 (18)	
C42	1.1760 (6)	0.6738 (6)	0.7281 (3)	0.066 (2)	
H42A	1.2159	0.6742	0.6976	0.099*	
H42B	1.1657	0.6030	0.7386	0.099*	
H42C	1.2251	0.7113	0.7510	0.099*	
C43	1.0738 (6)	1.0046 (6)	0.6782 (3)	0.070 (2)	
H43A	1.1400	1.0540	0.6766	0.106*	
H43B	1.0128	1.0299	0.6999	0.106*	
H43C	1.0379	0.9960	0.6470	0.106*	
C44	1.1211 (6)	0.9032 (6)	0.6954 (2)	0.055 (2)	
Fe1	0.35856 (7)	0.69453 (7)	0.53118 (3)	0.0321 (2)	
Fe2	0.60796 (7)	0.79888 (8)	0.77673 (3)	0.0371 (2)	
N1	0.7739 (5)	0.7822 (4)	0.59283 (18)	0.0423 (13)	
N2	0.6486 (4)	0.8042 (4)	0.59141 (17)	0.0405 (12)	
N3	0.7822 (4)	0.7929 (4)	0.46589 (17)	0.0413 (13)	
N4	0.6573 (4)	0.8160 (4)	0.46824 (18)	0.0455 (13)	
N5	1.0245 (5)	0.7520 (4)	0.84742 (18)	0.0368 (12)	
N6	0.9003 (4)	0.7320 (4)	0.84904 (17)	0.0333 (12)	
N7	1.0347 (4)	0.8261 (5)	0.70783 (17)	0.0443 (14)	
N8	0.9118 (4)	0.8515 (5)	0.70534 (18)	0.0478 (14)	
O1	0.9643 (4)	0.8490 (4)	0.5868 (2)	0.0741 (16)	
O2	0.9741 (4)	0.8544 (4)	0.4551 (2)	0.0821 (18)	
O3	1.2166 (4)	0.6856 (4)	0.85421 (17)	0.0568 (13)	
O4	1.2289 (4)	0.8845 (5)	0.7002 (2)	0.0858 (18)	

### Atomic displacement parameters (Å^2^)

**Table:**

	*U*^11^	*U*^22^	*U*^33^	*U*^12^	*U*^13^	*U*^23^
C1	0.048 (4)	0.049 (5)	0.097 (6)	−0.016 (4)	0.002 (4)	0.014 (4)
C2	0.040 (4)	0.073 (5)	0.050 (4)	−0.013 (4)	0.004 (3)	0.003 (4)
C3	0.029 (3)	0.064 (5)	0.058 (4)	0.003 (3)	−0.001 (3)	−0.005 (4)
C4	0.032 (3)	0.052 (5)	0.035 (3)	0.002 (3)	−0.004 (3)	−0.008 (3)
C5	0.032 (3)	0.042 (4)	0.041 (4)	−0.006 (3)	0.000 (3)	−0.005 (3)
C6	0.027 (3)	0.049 (4)	0.036 (3)	−0.005 (3)	−0.001 (2)	−0.006 (3)
C7	0.033 (3)	0.045 (4)	0.037 (3)	−0.001 (3)	−0.003 (3)	−0.003 (3)
C8	0.037 (3)	0.038 (4)	0.041 (4)	−0.003 (3)	0.001 (3)	0.002 (3)
C9	0.039 (4)	0.047 (4)	0.044 (4)	−0.005 (3)	0.004 (3)	0.006 (3)
C10	0.031 (4)	0.057 (5)	0.050 (4)	0.005 (3)	0.005 (3)	−0.009 (3)
C11	0.046 (4)	0.041 (4)	0.049 (4)	0.002 (3)	0.006 (3)	−0.013 (3)
C12	0.033 (3)	0.044 (4)	0.041 (4)	0.001 (3)	−0.003 (3)	−0.009 (3)
C13	0.036 (4)	0.061 (5)	0.039 (4)	−0.007 (3)	−0.007 (3)	−0.006 (3)
C14	0.029 (4)	0.071 (5)	0.054 (5)	0.006 (3)	−0.006 (3)	0.012 (4)
C15	0.041 (4)	0.044 (4)	0.056 (4)	0.001 (3)	0.002 (3)	0.011 (4)
C16	0.026 (3)	0.047 (4)	0.042 (4)	−0.005 (3)	0.005 (3)	0.006 (3)
C17	0.032 (4)	0.041 (4)	0.032 (3)	−0.005 (3)	0.004 (3)	0.001 (3)
C18	0.030 (3)	0.034 (3)	0.042 (4)	0.002 (3)	−0.002 (3)	−0.001 (3)
C19	0.029 (3)	0.053 (4)	0.035 (3)	0.001 (3)	0.002 (2)	0.002 (3)
C20	0.038 (4)	0.063 (5)	0.063 (5)	0.009 (3)	0.003 (3)	0.009 (4)
C21	0.054 (5)	0.043 (4)	0.107 (7)	−0.016 (4)	0.024 (4)	−0.004 (4)
C22	0.041 (4)	0.061 (5)	0.059 (5)	−0.018 (4)	0.009 (3)	0.003 (4)
C23	0.046 (4)	0.043 (4)	0.100 (6)	0.007 (3)	−0.009 (4)	0.007 (4)
C24	0.036 (4)	0.049 (4)	0.043 (4)	0.012 (3)	−0.004 (3)	0.000 (3)
C25	0.033 (3)	0.049 (4)	0.060 (4)	−0.006 (3)	0.004 (3)	−0.008 (3)
C26	0.036 (3)	0.039 (4)	0.032 (3)	−0.001 (3)	−0.002 (3)	−0.008 (3)
C27	0.039 (4)	0.033 (4)	0.043 (4)	0.002 (3)	−0.002 (3)	−0.005 (3)
C28	0.027 (3)	0.050 (4)	0.035 (3)	0.004 (3)	−0.004 (3)	−0.004 (3)
C29	0.028 (3)	0.035 (4)	0.035 (3)	−0.005 (3)	0.001 (2)	−0.006 (3)
C30	0.037 (4)	0.048 (4)	0.039 (4)	0.001 (3)	0.001 (3)	0.006 (3)
C31	0.021 (3)	0.065 (5)	0.047 (4)	−0.004 (3)	0.012 (3)	−0.001 (4)
C32	0.027 (3)	0.051 (4)	0.074 (5)	0.012 (3)	0.004 (3)	−0.002 (4)
C33	0.030 (3)	0.036 (4)	0.068 (5)	0.008 (3)	0.006 (3)	0.008 (3)
C34	0.038 (4)	0.083 (6)	0.052 (4)	−0.013 (4)	0.008 (3)	−0.025 (4)
C35	0.028 (4)	0.120 (7)	0.053 (5)	−0.028 (5)	0.002 (3)	−0.026 (5)
C36	0.029 (4)	0.142 (9)	0.041 (4)	0.002 (5)	−0.009 (3)	0.010 (5)
C37	0.035 (4)	0.108 (7)	0.039 (4)	−0.004 (4)	−0.004 (3)	0.023 (4)
C38	0.027 (4)	0.083 (5)	0.032 (4)	−0.009 (3)	0.004 (3)	−0.006 (3)
C39	0.025 (4)	0.079 (5)	0.032 (4)	0.001 (3)	0.003 (3)	−0.011 (3)
C40	0.033 (3)	0.055 (4)	0.044 (4)	−0.005 (3)	0.008 (3)	−0.007 (3)
C41	0.036 (4)	0.068 (5)	0.041 (4)	0.004 (4)	−0.002 (3)	−0.013 (4)
C42	0.034 (4)	0.088 (6)	0.076 (5)	0.011 (4)	0.011 (3)	−0.016 (5)
C43	0.046 (5)	0.071 (6)	0.094 (6)	−0.020 (4)	0.002 (4)	−0.002 (5)
C44	0.031 (4)	0.087 (6)	0.048 (4)	−0.012 (4)	0.007 (3)	−0.012 (4)
Fe1	0.0230 (4)	0.0347 (5)	0.0384 (5)	−0.0019 (4)	0.0011 (3)	−0.0006 (4)
Fe2	0.0185 (4)	0.0559 (6)	0.0369 (5)	−0.0008 (4)	−0.0001 (3)	0.0032 (5)
N1	0.034 (3)	0.056 (4)	0.037 (3)	−0.008 (3)	−0.006 (2)	−0.003 (3)
N2	0.029 (3)	0.048 (3)	0.045 (3)	−0.005 (3)	−0.004 (2)	−0.005 (3)
N3	0.028 (3)	0.049 (4)	0.047 (3)	−0.005 (3)	0.007 (2)	0.009 (3)
N4	0.032 (3)	0.053 (4)	0.052 (3)	0.003 (3)	0.005 (2)	0.006 (3)
N5	0.023 (3)	0.044 (3)	0.044 (3)	0.005 (2)	0.000 (2)	−0.002 (2)
N6	0.017 (3)	0.042 (3)	0.041 (3)	0.002 (2)	−0.004 (2)	−0.001 (2)
N7	0.015 (2)	0.076 (4)	0.042 (3)	−0.004 (3)	0.005 (2)	−0.001 (3)
N8	0.020 (3)	0.076 (4)	0.047 (3)	−0.002 (3)	0.004 (2)	−0.002 (3)
O1	0.033 (3)	0.087 (4)	0.103 (4)	−0.009 (3)	0.012 (3)	0.006 (3)
O2	0.027 (3)	0.089 (4)	0.130 (5)	−0.016 (3)	0.016 (3)	−0.001 (4)
O3	0.027 (2)	0.064 (3)	0.079 (3)	0.010 (2)	−0.002 (2)	0.004 (3)
O4	0.027 (3)	0.117 (5)	0.114 (5)	−0.007 (3)	0.008 (3)	0.005 (4)

### Geometric parameters (Å, °)

**Table:**

C1—C2	1.488 (9)	C23—H23B	0.9600
C1—H1A	0.9600	C23—H23C	0.9600
C1—H1B	0.9600	C24—O3	1.211 (7)
C1—H1C	0.9600	C24—N5	1.406 (7)
C2—O1	1.202 (7)	C25—C26	1.501 (8)
C2—N1	1.403 (8)	C25—H25A	0.9600
C3—C4	1.491 (8)	C25—H25B	0.9600
C3—H3A	0.9600	C25—H25C	0.9600
C3—H3B	0.9600	C25—H25D	0.9600
C3—H3C	0.9600	C25—H25E	0.9600
C3—H3D	0.9600	C25—H25F	0.9600
C3—H3E	0.9600	C26—C27	1.350 (8)
C3—H3F	0.9600	C26—N5	1.381 (7)
C4—C5	1.352 (8)	C27—C28	1.418 (8)
C4—N1	1.378 (8)	C27—H27	0.9300
C5—C6	1.414 (8)	C28—N6	1.314 (7)
C5—H5	0.9300	C28—C29	1.471 (7)
C6—N2	1.323 (7)	C29—C33	1.423 (8)
C6—C7	1.476 (8)	C29—C30	1.424 (8)
C7—C11	1.404 (8)	C29—Fe2	2.047 (5)
C7—C8	1.432 (8)	C30—C31	1.416 (8)
C7—Fe1	2.054 (5)	C30—Fe2	2.042 (6)
C8—C9	1.420 (8)	C30—H30	0.9300
C8—Fe1	2.057 (6)	C31—C32	1.387 (9)
C8—H8	0.9300	C31—Fe2	2.029 (5)
C9—C10	1.421 (8)	C31—H31	0.9300
C9—Fe1	2.051 (6)	C32—C33	1.413 (8)
C9—H9	0.9300	C32—Fe2	2.047 (6)
C10—C11	1.415 (9)	C32—H32	0.9300
C10—Fe1	2.047 (6)	C33—Fe2	2.030 (6)
C10—H10	0.9300	C33—H33	0.9300
C11—Fe1	2.025 (6)	C34—C38	1.415 (9)
C11—H11	0.9300	C34—C35	1.418 (9)
C12—C13	1.413 (8)	C34—Fe2	2.033 (7)
C12—C16	1.416 (8)	C34—H34	0.9300
C12—Fe1	2.047 (6)	C35—C36	1.373 (11)
C12—H12	0.9300	C35—Fe2	2.037 (7)
C13—C14	1.427 (9)	C35—H35	0.9300
C13—Fe1	2.042 (6)	C36—C37	1.420 (9)
C13—H13	0.9300	C36—Fe2	2.052 (6)
C14—C15	1.426 (9)	C36—H36	0.9300
C14—Fe1	2.046 (6)	C37—C38	1.434 (9)
C14—H14	0.9300	C37—Fe2	2.046 (6)
C15—C16	1.404 (8)	C37—H37	0.9300
C15—Fe1	2.043 (6)	C38—C39	1.465 (8)
C15—H15	0.9300	C38—Fe2	2.045 (6)
C16—C17	1.484 (8)	C39—N8	1.325 (8)
C16—Fe1	2.061 (6)	C39—C40	1.420 (9)
C17—N4	1.313 (7)	C40—C41	1.352 (8)
C17—C18	1.423 (8)	C40—H40	0.9300
C18—C19	1.357 (8)	C41—N7	1.373 (8)
C18—H18	0.9300	C41—C42	1.507 (9)
C19—N3	1.370 (8)	C42—H42A	0.9600
C19—C20	1.477 (8)	C42—H42B	0.9600
C20—H20A	0.9600	C42—H42C	0.9600
C20—H20B	0.9600	C43—C44	1.476 (10)
C20—H20C	0.9600	C43—H43A	0.9600
C21—C22	1.485 (9)	C43—H43B	0.9600
C21—H21A	0.9600	C43—H43C	0.9600
C21—H21B	0.9600	C44—O4	1.206 (7)
C21—H21C	0.9600	C44—N7	1.410 (8)
C22—O2	1.218 (8)	N1—N2	1.396 (7)
C22—N3	1.418 (8)	N3—N4	1.395 (6)
C23—C24	1.481 (9)	N5—N6	1.378 (6)
C23—H23A	0.9600	N7—N8	1.380 (6)
			
C2—C1—H1A	109.5	C30—C31—Fe2	70.1 (3)
C2—C1—H1B	109.5	C32—C31—H31	125.5
H1A—C1—H1B	109.5	C30—C31—H31	125.5
C2—C1—H1C	109.5	Fe2—C31—H31	125.1
H1A—C1—H1C	109.5	C31—C32—C33	108.0 (5)
H1B—C1—H1C	109.5	C31—C32—Fe2	69.4 (3)
O1—C2—N1	119.6 (7)	C33—C32—Fe2	69.1 (4)
O1—C2—C1	124.4 (7)	C31—C32—H32	126.0
N1—C2—C1	116.0 (6)	C33—C32—H32	126.0
C4—C3—H3A	109.5	Fe2—C32—H32	127.0
C4—C3—H3B	109.5	C32—C33—C29	108.7 (6)
H3A—C3—H3B	109.5	C32—C33—Fe2	70.4 (4)
C4—C3—H3C	109.5	C29—C33—Fe2	70.2 (3)
H3A—C3—H3C	109.5	C32—C33—H33	125.7
H3B—C3—H3C	109.5	C29—C33—H33	125.7
C4—C3—H3D	109.5	Fe2—C33—H33	125.4
H3A—C3—H3D	141.1	C38—C34—C35	107.8 (7)
H3B—C3—H3D	56.3	C38—C34—Fe2	70.2 (4)
H3C—C3—H3D	56.3	C35—C34—Fe2	69.8 (4)
C4—C3—H3E	109.5	C38—C34—H34	126.1
H3A—C3—H3E	56.3	C35—C34—H34	126.1
H3B—C3—H3E	141.1	Fe2—C34—H34	125.5
H3C—C3—H3E	56.3	C36—C35—C34	109.0 (7)
H3D—C3—H3E	109.5	C36—C35—Fe2	71.0 (4)
C4—C3—H3F	109.5	C34—C35—Fe2	69.5 (4)
H3A—C3—H3F	56.3	C36—C35—H35	125.5
H3B—C3—H3F	56.3	C34—C35—H35	125.5
H3C—C3—H3F	141.1	Fe2—C35—H35	125.6
H3D—C3—H3F	109.5	C35—C36—C37	108.9 (7)
H3E—C3—H3F	109.5	C35—C36—Fe2	69.8 (4)
C5—C4—N1	105.1 (5)	C37—C36—Fe2	69.5 (4)
C5—C4—C3	130.6 (6)	C35—C36—H36	125.6
N1—C4—C3	124.3 (6)	C37—C36—H36	125.6
C4—C5—C6	107.0 (6)	Fe2—C36—H36	126.7
C4—C5—H5	126.5	C36—C37—C38	107.1 (7)
C6—C5—H5	126.5	C36—C37—Fe2	70.0 (4)
N2—C6—C5	112.2 (5)	C38—C37—Fe2	69.5 (4)
N2—C6—C7	119.2 (6)	C36—C37—H37	126.4
C5—C6—C7	128.6 (6)	C38—C37—H37	126.4
C11—C7—C8	107.2 (5)	Fe2—C37—H37	125.7
C11—C7—C6	127.2 (6)	C34—C38—C37	107.2 (6)
C8—C7—C6	125.6 (6)	C34—C38—C39	127.4 (7)
C11—C7—Fe1	68.8 (3)	C37—C38—C39	125.3 (6)
C8—C7—Fe1	69.7 (3)	C34—C38—Fe2	69.3 (4)
C6—C7—Fe1	124.8 (4)	C37—C38—Fe2	69.5 (4)
C9—C8—C7	108.1 (5)	C39—C38—Fe2	128.3 (4)
C9—C8—Fe1	69.6 (3)	N8—C39—C40	111.9 (6)
C7—C8—Fe1	69.5 (3)	N8—C39—C38	120.4 (6)
C9—C8—H8	125.9	C40—C39—C38	127.7 (7)
C7—C8—H8	125.9	C41—C40—C39	105.6 (6)
Fe1—C8—H8	126.6	C41—C40—H40	127.2
C8—C9—C10	107.8 (6)	C39—C40—H40	127.2
C8—C9—Fe1	70.0 (3)	C40—C41—N7	106.8 (6)
C10—C9—Fe1	69.6 (4)	C40—C41—C42	129.1 (7)
C8—C9—H9	126.1	N7—C41—C42	124.1 (6)
C10—C9—H9	126.1	C41—C42—H42A	109.5
Fe1—C9—H9	125.9	C41—C42—H42B	109.5
C11—C10—C9	107.6 (6)	H42A—C42—H42B	109.5
C11—C10—Fe1	68.8 (4)	C41—C42—H42C	109.5
C9—C10—Fe1	69.9 (4)	H42A—C42—H42C	109.5
C11—C10—H10	126.2	H42B—C42—H42C	109.5
C9—C10—H10	126.2	C44—C43—H43A	109.5
Fe1—C10—H10	126.6	C44—C43—H43B	109.5
C7—C11—C10	109.3 (6)	H43A—C43—H43B	109.5
C7—C11—Fe1	71.0 (3)	C44—C43—H43C	109.5
C10—C11—Fe1	70.5 (4)	H43A—C43—H43C	109.5
C7—C11—H11	125.3	H43B—C43—H43C	109.5
C10—C11—H11	125.3	O4—C44—N7	119.1 (7)
Fe1—C11—H11	124.8	O4—C44—C43	123.3 (7)
C13—C12—C16	108.2 (6)	N7—C44—C43	117.6 (6)
C13—C12—Fe1	69.6 (3)	C11—Fe1—C15	105.8 (3)
C16—C12—Fe1	70.4 (3)	C11—Fe1—C13	157.5 (3)
C13—C12—H12	125.9	C15—Fe1—C13	68.3 (3)
C16—C12—H12	125.9	C11—Fe1—C14	120.6 (3)
Fe1—C12—H12	125.7	C15—Fe1—C14	40.8 (2)
C12—C13—C14	108.1 (6)	C13—Fe1—C14	40.9 (2)
C12—C13—Fe1	70.0 (3)	C11—Fe1—C12	159.4 (3)
C14—C13—Fe1	69.7 (4)	C15—Fe1—C12	67.8 (3)
C12—C13—H13	125.9	C13—Fe1—C12	40.4 (2)
C14—C13—H13	125.9	C14—Fe1—C12	68.4 (3)
Fe1—C13—H13	125.9	C11—Fe1—C10	40.6 (2)
C15—C14—C13	106.9 (6)	C15—Fe1—C10	120.7 (3)
C15—C14—Fe1	69.5 (4)	C13—Fe1—C10	122.2 (3)
C13—C14—Fe1	69.4 (4)	C14—Fe1—C10	105.1 (3)
C15—C14—H14	126.5	C12—Fe1—C10	159.6 (3)
C13—C14—H14	126.5	C11—Fe1—C9	68.3 (3)
Fe1—C14—H14	126.2	C15—Fe1—C9	157.1 (3)
C16—C15—C14	108.6 (6)	C13—Fe1—C9	108.2 (3)
C16—C15—Fe1	70.7 (4)	C14—Fe1—C9	121.6 (3)
C14—C15—Fe1	69.7 (4)	C12—Fe1—C9	125.1 (3)
C16—C15—H15	125.7	C10—Fe1—C9	40.6 (2)
C14—C15—H15	125.7	C11—Fe1—C7	40.3 (2)
Fe1—C15—H15	125.5	C15—Fe1—C7	122.2 (3)
C15—C16—C12	108.1 (5)	C13—Fe1—C7	161.1 (3)
C15—C16—C17	126.4 (6)	C14—Fe1—C7	157.1 (3)
C12—C16—C17	125.5 (6)	C12—Fe1—C7	125.2 (2)
C15—C16—Fe1	69.3 (4)	C10—Fe1—C7	68.2 (2)
C12—C16—Fe1	69.3 (3)	C9—Fe1—C7	68.4 (2)
C17—C16—Fe1	128.7 (4)	C11—Fe1—C8	68.0 (2)
N4—C17—C18	112.1 (6)	C15—Fe1—C8	159.8 (2)
N4—C17—C16	120.6 (6)	C13—Fe1—C8	124.7 (3)
C18—C17—C16	127.2 (6)	C14—Fe1—C8	159.0 (3)
C19—C18—C17	106.6 (6)	C12—Fe1—C8	110.8 (2)
C19—C18—H18	126.7	C10—Fe1—C8	68.0 (3)
C17—C18—H18	126.7	C9—Fe1—C8	40.4 (2)
C18—C19—N3	105.1 (5)	C7—Fe1—C8	40.8 (2)
C18—C19—C20	129.4 (6)	C11—Fe1—C16	122.4 (3)
N3—C19—C20	125.4 (6)	C15—Fe1—C16	40.0 (2)
C19—C20—H20A	109.5	C13—Fe1—C16	67.9 (2)
C19—C20—H20B	109.5	C14—Fe1—C16	68.1 (3)
H20A—C20—H20B	109.5	C12—Fe1—C16	40.3 (2)
C19—C20—H20C	109.5	C10—Fe1—C16	157.1 (3)
H20A—C20—H20C	109.5	C9—Fe1—C16	161.6 (3)
H20B—C20—H20C	109.5	C7—Fe1—C16	109.0 (2)
C22—C21—H21A	109.5	C8—Fe1—C16	125.8 (2)
C22—C21—H21B	109.5	C31—Fe2—C33	67.8 (3)
H21A—C21—H21B	109.5	C31—Fe2—C34	126.4 (3)
C22—C21—H21C	109.5	C33—Fe2—C34	155.6 (2)
H21A—C21—H21C	109.5	C31—Fe2—C35	107.9 (3)
H21B—C21—H21C	109.5	C33—Fe2—C35	162.1 (3)
O2—C22—N3	117.7 (7)	C34—Fe2—C35	40.8 (3)
O2—C22—C21	124.8 (6)	C31—Fe2—C30	40.7 (2)
N3—C22—C21	117.6 (6)	C33—Fe2—C30	68.2 (3)
C24—C23—H23A	109.5	C34—Fe2—C30	108.4 (3)
C24—C23—H23B	109.5	C35—Fe2—C30	120.8 (3)
H23A—C23—H23B	109.5	C31—Fe2—C38	163.9 (3)
C24—C23—H23C	109.5	C33—Fe2—C38	120.7 (3)
H23A—C23—H23C	109.5	C34—Fe2—C38	40.6 (3)
H23B—C23—H23C	109.5	C35—Fe2—C38	68.2 (3)
O3—C24—N5	119.9 (6)	C30—Fe2—C38	126.7 (3)
O3—C24—C23	124.1 (6)	C31—Fe2—C37	153.5 (3)
N5—C24—C23	116.0 (6)	C33—Fe2—C37	107.9 (3)
C26—C25—H25A	109.5	C34—Fe2—C37	68.4 (3)
C26—C25—H25B	109.5	C35—Fe2—C37	67.6 (3)
H25A—C25—H25B	109.5	C30—Fe2—C37	164.3 (3)
C26—C25—H25C	109.5	C38—Fe2—C37	41.0 (3)
H25A—C25—H25C	109.5	C31—Fe2—C29	68.6 (2)
H25B—C25—H25C	109.5	C33—Fe2—C29	40.9 (2)
C26—C25—H25D	109.5	C34—Fe2—C29	120.6 (3)
H25A—C25—H25D	141.1	C35—Fe2—C29	155.6 (3)
H25B—C25—H25D	56.3	C30—Fe2—C29	40.8 (2)
H25C—C25—H25D	56.3	C38—Fe2—C29	108.1 (2)
C26—C25—H25E	109.5	C37—Fe2—C29	126.4 (3)
H25A—C25—H25E	56.3	C31—Fe2—C32	39.8 (2)
H25B—C25—H25E	141.1	C33—Fe2—C32	40.5 (2)
H25C—C25—H25E	56.3	C34—Fe2—C32	162.6 (3)
H25D—C25—H25E	109.5	C35—Fe2—C32	125.2 (3)
C26—C25—H25F	109.5	C30—Fe2—C32	67.8 (3)
H25A—C25—H25F	56.3	C38—Fe2—C32	155.2 (3)
H25B—C25—H25F	56.3	C37—Fe2—C32	119.8 (3)
H25C—C25—H25F	141.1	C29—Fe2—C32	68.5 (2)
H25D—C25—H25F	109.5	C31—Fe2—C36	119.4 (3)
H25E—C25—H25F	109.5	C33—Fe2—C36	126.3 (3)
C27—C26—N5	106.0 (5)	C34—Fe2—C36	67.6 (3)
C27—C26—C25	129.5 (6)	C35—Fe2—C36	39.2 (3)
N5—C26—C25	124.5 (5)	C30—Fe2—C36	154.0 (3)
C26—C27—C28	106.1 (6)	C38—Fe2—C36	68.2 (3)
C26—C27—H27	126.9	C37—Fe2—C36	40.6 (3)
C28—C27—H27	126.9	C29—Fe2—C36	163.9 (3)
N6—C28—C27	111.9 (5)	C32—Fe2—C36	107.8 (3)
N6—C28—C29	120.5 (5)	C4—N1—N2	112.9 (5)
C27—C28—C29	127.7 (6)	C4—N1—C2	129.0 (6)
C33—C29—C30	106.6 (5)	N2—N1—C2	118.1 (6)
C33—C29—C28	126.5 (5)	C6—N2—N1	102.8 (5)
C30—C29—C28	126.9 (6)	C19—N3—N4	113.1 (5)
C33—C29—Fe2	69.0 (3)	C19—N3—C22	128.3 (5)
C30—C29—Fe2	69.4 (3)	N4—N3—C22	118.5 (6)
C28—C29—Fe2	125.5 (4)	C17—N4—N3	103.1 (5)
C31—C30—C29	107.9 (6)	N6—N5—C26	111.8 (5)
C31—C30—Fe2	69.2 (3)	N6—N5—C24	119.0 (5)
C29—C30—Fe2	69.8 (3)	C26—N5—C24	129.1 (5)
C31—C30—H30	126.1	C28—N6—N5	104.2 (5)
C29—C30—H30	126.1	C41—N7—N8	111.7 (5)
Fe2—C30—H30	126.6	C41—N7—C44	130.1 (6)
C32—C31—C30	108.9 (5)	N8—N7—C44	118.3 (6)
C32—C31—Fe2	70.8 (4)	C39—N8—N7	104.0 (5)
			
N1—C4—C5—C6	0.2 (7)	C7—C8—Fe1—C14	−156.2 (7)
C3—C4—C5—C6	−178.9 (6)	C9—C8—Fe1—C12	−120.2 (4)
C4—C5—C6—N2	−0.8 (7)	C7—C8—Fe1—C12	120.2 (4)
C4—C5—C6—C7	179.3 (6)	C9—C8—Fe1—C10	37.9 (4)
N2—C6—C7—C11	−3.2 (9)	C7—C8—Fe1—C10	−81.7 (4)
C5—C6—C7—C11	176.6 (6)	C7—C8—Fe1—C9	−119.6 (5)
N2—C6—C7—C8	174.1 (5)	C9—C8—Fe1—C7	119.6 (5)
C5—C6—C7—C8	−6.1 (10)	C9—C8—Fe1—C16	−163.1 (4)
N2—C6—C7—Fe1	85.3 (7)	C7—C8—Fe1—C16	77.3 (4)
C5—C6—C7—Fe1	−94.8 (7)	C15—C16—Fe1—C11	−75.3 (4)
C11—C7—C8—C9	−0.2 (7)	C12—C16—Fe1—C11	164.9 (4)
C6—C7—C8—C9	−178.0 (5)	C17—C16—Fe1—C11	45.3 (7)
Fe1—C7—C8—C9	−59.0 (4)	C12—C16—Fe1—C15	−119.8 (5)
C11—C7—C8—Fe1	58.8 (4)	C17—C16—Fe1—C15	120.6 (8)
C6—C7—C8—Fe1	−119.0 (6)	C15—C16—Fe1—C13	82.1 (4)
C7—C8—C9—C10	−0.5 (7)	C12—C16—Fe1—C13	−37.7 (4)
Fe1—C8—C9—C10	−59.5 (5)	C17—C16—Fe1—C13	−157.3 (7)
C7—C8—C9—Fe1	59.0 (4)	C15—C16—Fe1—C14	37.9 (4)
C8—C9—C10—C11	1.1 (8)	C12—C16—Fe1—C14	−81.9 (4)
Fe1—C9—C10—C11	−58.7 (5)	C17—C16—Fe1—C14	158.5 (7)
C8—C9—C10—Fe1	59.8 (4)	C15—C16—Fe1—C12	119.8 (5)
C8—C7—C11—C10	0.9 (7)	C17—C16—Fe1—C12	−119.6 (7)
C6—C7—C11—C10	178.6 (6)	C15—C16—Fe1—C10	−38.9 (8)
Fe1—C7—C11—C10	60.3 (5)	C12—C16—Fe1—C10	−158.7 (6)
C8—C7—C11—Fe1	−59.4 (4)	C17—C16—Fe1—C10	81.7 (9)
C6—C7—C11—Fe1	118.3 (6)	C15—C16—Fe1—C9	163.3 (7)
C9—C10—C11—C7	−1.3 (8)	C12—C16—Fe1—C9	43.5 (9)
Fe1—C10—C11—C7	−60.6 (4)	C17—C16—Fe1—C9	−76.1 (10)
C9—C10—C11—Fe1	59.3 (5)	C15—C16—Fe1—C7	−117.8 (4)
C16—C12—C13—C14	−0.5 (7)	C12—C16—Fe1—C7	122.3 (4)
Fe1—C12—C13—C14	59.5 (5)	C17—C16—Fe1—C7	2.8 (6)
C16—C12—C13—Fe1	−60.0 (4)	C15—C16—Fe1—C8	−160.2 (4)
C12—C13—C14—C15	−0.1 (8)	C12—C16—Fe1—C8	80.0 (4)
Fe1—C13—C14—C15	59.6 (5)	C17—C16—Fe1—C8	−39.6 (7)
C12—C13—C14—Fe1	−59.7 (4)	C32—C31—Fe2—C33	37.6 (4)
C13—C14—C15—C16	0.7 (8)	C30—C31—Fe2—C33	−81.8 (4)
Fe1—C14—C15—C16	60.2 (5)	C32—C31—Fe2—C34	−165.3 (4)
C13—C14—C15—Fe1	−59.6 (5)	C30—C31—Fe2—C34	75.3 (5)
C14—C15—C16—C12	−1.0 (7)	C32—C31—Fe2—C35	−123.9 (4)
Fe1—C15—C16—C12	58.6 (4)	C30—C31—Fe2—C35	116.7 (4)
C14—C15—C16—C17	176.9 (6)	C32—C31—Fe2—C30	119.4 (5)
Fe1—C15—C16—C17	−123.5 (6)	C32—C31—Fe2—C38	162.9 (8)
C14—C15—C16—Fe1	−59.6 (5)	C30—C31—Fe2—C38	43.5 (11)
C13—C12—C16—C15	1.0 (7)	C32—C31—Fe2—C37	−48.3 (8)
Fe1—C12—C16—C15	−58.6 (4)	C30—C31—Fe2—C37	−167.7 (6)
C13—C12—C16—C17	−177.0 (5)	C32—C31—Fe2—C29	81.7 (4)
Fe1—C12—C16—C17	123.5 (6)	C30—C31—Fe2—C29	−37.7 (4)
C13—C12—C16—Fe1	59.6 (4)	C30—C31—Fe2—C32	−119.4 (5)
C15—C16—C17—N4	−6.6 (10)	C32—C31—Fe2—C36	−82.7 (5)
C12—C16—C17—N4	170.9 (6)	C30—C31—Fe2—C36	157.9 (5)
Fe1—C16—C17—N4	−98.2 (7)	C32—C33—Fe2—C31	−36.9 (4)
C15—C16—C17—C18	177.7 (6)	C29—C33—Fe2—C31	82.4 (4)
C12—C16—C17—C18	−4.8 (10)	C32—C33—Fe2—C34	−167.6 (6)
Fe1—C16—C17—C18	86.1 (8)	C29—C33—Fe2—C34	−48.3 (8)
N4—C17—C18—C19	0.6 (7)	C32—C33—Fe2—C35	42.6 (11)
C16—C17—C18—C19	176.6 (6)	C29—C33—Fe2—C35	161.9 (8)
C17—C18—C19—N3	−0.1 (6)	C32—C33—Fe2—C30	−81.0 (4)
C17—C18—C19—C20	−178.8 (6)	C29—C33—Fe2—C30	38.4 (3)
N5—C26—C27—C28	−0.4 (7)	C32—C33—Fe2—C38	158.3 (4)
C25—C26—C27—C28	179.4 (6)	C29—C33—Fe2—C38	−82.3 (4)
C26—C27—C28—N6	0.1 (7)	C32—C33—Fe2—C37	115.2 (4)
C26—C27—C28—C29	−179.0 (6)	C29—C33—Fe2—C37	−125.4 (4)
N6—C28—C29—C33	−174.9 (6)	C32—C33—Fe2—C29	−119.3 (5)
C27—C28—C29—C33	4.2 (10)	C29—C33—Fe2—C32	119.3 (5)
N6—C28—C29—C30	3.6 (9)	C32—C33—Fe2—C36	74.1 (5)
C27—C28—C29—C30	−177.3 (6)	C29—C33—Fe2—C36	−166.6 (4)
N6—C28—C29—Fe2	−86.1 (7)	C38—C34—Fe2—C31	−167.0 (4)
C27—C28—C29—Fe2	92.9 (7)	C35—C34—Fe2—C31	74.4 (6)
C33—C29—C30—C31	0.4 (7)	C38—C34—Fe2—C33	−47.7 (9)
C28—C29—C30—C31	−178.4 (5)	C35—C34—Fe2—C33	−166.3 (6)
Fe2—C29—C30—C31	−58.9 (4)	C38—C34—Fe2—C35	118.6 (7)
C33—C29—C30—Fe2	59.2 (4)	C38—C34—Fe2—C30	−125.3 (4)
C28—C29—C30—Fe2	−119.6 (6)	C35—C34—Fe2—C30	116.0 (5)
C29—C30—C31—C32	−1.1 (7)	C35—C34—Fe2—C38	−118.6 (7)
Fe2—C30—C31—C32	−60.4 (5)	C38—C34—Fe2—C37	38.3 (4)
C29—C30—C31—Fe2	59.3 (4)	C35—C34—Fe2—C37	−80.3 (5)
C30—C31—C32—C33	1.5 (8)	C38—C34—Fe2—C29	−82.3 (5)
Fe2—C31—C32—C33	−58.5 (5)	C35—C34—Fe2—C29	159.1 (4)
C30—C31—C32—Fe2	60.0 (4)	C38—C34—Fe2—C32	160.1 (8)
C31—C32—C33—C29	−1.3 (8)	C35—C34—Fe2—C32	41.5 (12)
Fe2—C32—C33—C29	−60.0 (4)	C38—C34—Fe2—C36	82.2 (5)
C31—C32—C33—Fe2	58.7 (5)	C35—C34—Fe2—C36	−36.4 (4)
C30—C29—C33—C32	0.5 (7)	C36—C35—Fe2—C31	114.8 (4)
C28—C29—C33—C32	179.3 (6)	C34—C35—Fe2—C31	−125.4 (5)
Fe2—C29—C33—C32	60.1 (5)	C36—C35—Fe2—C33	41.7 (11)
C30—C29—C33—Fe2	−59.5 (4)	C34—C35—Fe2—C33	161.5 (8)
C28—C29—C33—Fe2	119.3 (6)	C36—C35—Fe2—C34	−119.8 (6)
C38—C34—C35—C36	0.1 (8)	C36—C35—Fe2—C30	157.5 (4)
Fe2—C34—C35—C36	60.2 (5)	C34—C35—Fe2—C30	−82.7 (5)
C38—C34—C35—Fe2	−60.1 (5)	C36—C35—Fe2—C38	−81.8 (5)
C34—C35—C36—C37	−0.6 (8)	C34—C35—Fe2—C38	38.0 (4)
Fe2—C35—C36—C37	58.6 (5)	C36—C35—Fe2—C37	−37.3 (4)
C34—C35—C36—Fe2	−59.3 (5)	C34—C35—Fe2—C37	82.4 (5)
C35—C36—C37—C38	0.9 (8)	C36—C35—Fe2—C29	−167.7 (5)
Fe2—C36—C37—C38	59.8 (5)	C34—C35—Fe2—C29	−48.0 (8)
C35—C36—C37—Fe2	−58.8 (5)	C36—C35—Fe2—C32	74.3 (5)
C35—C34—C38—C37	0.5 (7)	C34—C35—Fe2—C32	−166.0 (4)
Fe2—C34—C38—C37	−59.4 (5)	C34—C35—Fe2—C36	119.8 (6)
C35—C34—C38—C39	−177.1 (6)	C29—C30—Fe2—C31	−119.3 (5)
Fe2—C34—C38—C39	123.1 (6)	C31—C30—Fe2—C33	80.9 (4)
C35—C34—C38—Fe2	59.9 (5)	C29—C30—Fe2—C33	−38.4 (3)
C36—C37—C38—C34	−0.9 (8)	C31—C30—Fe2—C34	−124.8 (4)
Fe2—C37—C38—C34	59.2 (4)	C29—C30—Fe2—C34	115.8 (4)
C36—C37—C38—C39	176.8 (6)	C31—C30—Fe2—C35	−81.8 (5)
Fe2—C37—C38—C39	−123.2 (6)	C29—C30—Fe2—C35	158.9 (4)
C36—C37—C38—Fe2	−60.1 (5)	C31—C30—Fe2—C38	−166.3 (4)
C34—C38—C39—N8	177.1 (6)	C29—C30—Fe2—C38	74.4 (4)
C37—C38—C39—N8	−0.1 (10)	C31—C30—Fe2—C37	159.5 (10)
Fe2—C38—C39—N8	−90.8 (8)	C29—C30—Fe2—C37	40.1 (12)
C34—C38—C39—C40	−1.7 (11)	C31—C30—Fe2—C29	119.3 (5)
C37—C38—C39—C40	−178.8 (6)	C31—C30—Fe2—C32	37.0 (4)
Fe2—C38—C39—C40	90.5 (8)	C29—C30—Fe2—C32	−82.3 (4)
N8—C39—C40—C41	−1.1 (7)	C31—C30—Fe2—C36	−48.4 (9)
C38—C39—C40—C41	177.7 (6)	C29—C30—Fe2—C36	−167.7 (7)
C39—C40—C41—N7	1.4 (7)	C34—C38—Fe2—C31	40.7 (11)
C39—C40—C41—C42	−177.6 (6)	C37—C38—Fe2—C31	159.4 (8)
C7—C11—Fe1—C15	−121.5 (4)	C39—C38—Fe2—C31	−81.2 (12)
C10—C11—Fe1—C15	118.9 (4)	C34—C38—Fe2—C33	159.2 (4)
C7—C11—Fe1—C13	167.1 (6)	C37—C38—Fe2—C33	−82.1 (4)
C10—C11—Fe1—C13	47.6 (9)	C39—C38—Fe2—C33	37.2 (8)
C7—C11—Fe1—C14	−163.3 (4)	C37—C38—Fe2—C34	118.7 (6)
C10—C11—Fe1—C14	77.2 (5)	C39—C38—Fe2—C34	−121.9 (9)
C7—C11—Fe1—C12	−52.6 (9)	C34—C38—Fe2—C35	−38.1 (5)
C10—C11—Fe1—C12	−172.1 (7)	C37—C38—Fe2—C35	80.5 (5)
C7—C11—Fe1—C10	119.5 (6)	C39—C38—Fe2—C35	−160.1 (8)
C7—C11—Fe1—C9	81.8 (4)	C34—C38—Fe2—C30	74.8 (5)
C10—C11—Fe1—C9	−37.7 (4)	C37—C38—Fe2—C30	−166.6 (4)
C10—C11—Fe1—C7	−119.5 (6)	C39—C38—Fe2—C30	−47.2 (8)
C7—C11—Fe1—C8	38.1 (4)	C34—C38—Fe2—C37	−118.7 (6)
C10—C11—Fe1—C8	−81.4 (4)	C39—C38—Fe2—C37	119.4 (8)
C7—C11—Fe1—C16	−81.3 (4)	C34—C38—Fe2—C29	116.2 (4)
C10—C11—Fe1—C16	159.2 (4)	C37—C38—Fe2—C29	−125.1 (4)
C16—C15—Fe1—C11	121.9 (4)	C39—C38—Fe2—C29	−5.8 (7)
C14—C15—Fe1—C11	−118.7 (4)	C34—C38—Fe2—C32	−166.0 (6)
C16—C15—Fe1—C13	−81.0 (4)	C37—C38—Fe2—C32	−47.3 (8)
C14—C15—Fe1—C13	38.3 (4)	C39—C38—Fe2—C32	72.1 (10)
C16—C15—Fe1—C14	−119.4 (6)	C34—C38—Fe2—C36	−80.5 (5)
C16—C15—Fe1—C12	−37.3 (4)	C37—C38—Fe2—C36	38.1 (5)
C14—C15—Fe1—C12	82.1 (4)	C39—C38—Fe2—C36	157.5 (8)
C16—C15—Fe1—C10	163.5 (4)	C36—C37—Fe2—C31	−49.2 (9)
C14—C15—Fe1—C10	−77.2 (5)	C38—C37—Fe2—C31	−167.4 (6)
C16—C15—Fe1—C9	−166.5 (6)	C36—C37—Fe2—C33	−125.4 (5)
C14—C15—Fe1—C9	−47.1 (9)	C38—C37—Fe2—C33	116.5 (4)
C16—C15—Fe1—C7	81.3 (4)	C36—C37—Fe2—C34	80.3 (5)
C14—C15—Fe1—C7	−159.3 (4)	C38—C37—Fe2—C34	−37.9 (4)
C16—C15—Fe1—C8	52.9 (9)	C36—C37—Fe2—C35	36.2 (5)
C14—C15—Fe1—C8	172.2 (7)	C38—C37—Fe2—C35	−82.0 (5)
C14—C15—Fe1—C16	119.4 (6)	C36—C37—Fe2—C30	161.6 (10)
C12—C13—Fe1—C11	159.7 (6)	C38—C37—Fe2—C30	43.5 (13)
C14—C13—Fe1—C11	40.6 (9)	C36—C37—Fe2—C38	118.2 (7)
C12—C13—Fe1—C15	80.9 (4)	C36—C37—Fe2—C29	−166.8 (5)
C14—C13—Fe1—C15	−38.3 (4)	C38—C37—Fe2—C29	75.0 (5)
C12—C13—Fe1—C14	119.2 (6)	C36—C37—Fe2—C32	−82.7 (6)
C14—C13—Fe1—C12	−119.2 (6)	C38—C37—Fe2—C32	159.2 (4)
C12—C13—Fe1—C10	−165.7 (4)	C38—C37—Fe2—C36	−118.2 (7)
C14—C13—Fe1—C10	75.2 (5)	C33—C29—Fe2—C31	−80.4 (4)
C12—C13—Fe1—C9	−123.2 (4)	C30—C29—Fe2—C31	37.7 (4)
C14—C13—Fe1—C9	117.6 (4)	C28—C29—Fe2—C31	159.0 (6)
C12—C13—Fe1—C7	−46.7 (9)	C30—C29—Fe2—C33	118.1 (5)
C14—C13—Fe1—C7	−165.9 (7)	C28—C29—Fe2—C33	−120.6 (6)
C12—C13—Fe1—C8	−81.5 (4)	C33—C29—Fe2—C34	159.0 (4)
C14—C13—Fe1—C8	159.3 (4)	C30—C29—Fe2—C34	−82.9 (4)
C12—C13—Fe1—C16	37.6 (4)	C28—C29—Fe2—C34	38.5 (6)
C14—C13—Fe1—C16	−81.6 (4)	C33—C29—Fe2—C35	−166.7 (6)
C15—C14—Fe1—C11	78.5 (5)	C30—C29—Fe2—C35	−48.6 (7)
C13—C14—Fe1—C11	−163.2 (4)	C28—C29—Fe2—C35	72.8 (8)
C13—C14—Fe1—C15	118.2 (6)	C33—C29—Fe2—C30	−118.1 (5)
C15—C14—Fe1—C13	−118.2 (6)	C28—C29—Fe2—C30	121.4 (7)
C15—C14—Fe1—C12	−80.7 (4)	C33—C29—Fe2—C38	116.3 (4)
C13—C14—Fe1—C12	37.5 (4)	C30—C29—Fe2—C38	−125.6 (4)
C15—C14—Fe1—C10	119.7 (4)	C28—C29—Fe2—C38	−4.3 (6)
C13—C14—Fe1—C10	−122.1 (4)	C33—C29—Fe2—C37	74.4 (5)
C15—C14—Fe1—C9	160.5 (4)	C30—C29—Fe2—C37	−167.5 (4)
C13—C14—Fe1—C9	−81.3 (5)	C28—C29—Fe2—C37	−46.1 (6)
C15—C14—Fe1—C7	50.0 (8)	C33—C29—Fe2—C32	−37.5 (4)
C13—C14—Fe1—C7	168.3 (5)	C30—C29—Fe2—C32	80.6 (4)
C15—C14—Fe1—C8	−172.5 (6)	C28—C29—Fe2—C32	−158.1 (6)
C13—C14—Fe1—C8	−54.3 (9)	C33—C29—Fe2—C36	42.3 (11)
C15—C14—Fe1—C16	−37.2 (4)	C30—C29—Fe2—C36	160.3 (9)
C13—C14—Fe1—C16	81.1 (4)	C28—C29—Fe2—C36	−78.3 (11)
C13—C12—Fe1—C11	−157.9 (7)	C33—C32—Fe2—C31	119.7 (6)
C16—C12—Fe1—C11	−38.8 (9)	C31—C32—Fe2—C33	−119.7 (6)
C13—C12—Fe1—C15	−82.1 (4)	C31—C32—Fe2—C34	43.0 (11)
C16—C12—Fe1—C15	37.0 (3)	C33—C32—Fe2—C34	162.8 (9)
C16—C12—Fe1—C13	119.1 (5)	C31—C32—Fe2—C35	75.0 (5)
C13—C12—Fe1—C14	−37.9 (4)	C33—C32—Fe2—C35	−165.3 (4)
C16—C12—Fe1—C14	81.2 (4)	C31—C32—Fe2—C30	−37.9 (4)
C13—C12—Fe1—C10	37.0 (9)	C33—C32—Fe2—C30	81.9 (4)
C16—C12—Fe1—C10	156.1 (7)	C31—C32—Fe2—C38	−168.8 (5)
C13—C12—Fe1—C9	76.3 (5)	C33—C32—Fe2—C38	−49.1 (8)
C16—C12—Fe1—C9	−164.6 (4)	C31—C32—Fe2—C37	157.4 (4)
C13—C12—Fe1—C7	163.2 (4)	C33—C32—Fe2—C37	−82.9 (4)
C16—C12—Fe1—C7	−77.7 (4)	C31—C32—Fe2—C29	−81.9 (4)
C13—C12—Fe1—C8	119.6 (4)	C33—C32—Fe2—C29	37.8 (4)
C16—C12—Fe1—C8	−121.3 (4)	C31—C32—Fe2—C36	114.8 (4)
C13—C12—Fe1—C16	−119.1 (5)	C33—C32—Fe2—C36	−125.5 (4)
C9—C10—Fe1—C11	−119.1 (6)	C35—C36—Fe2—C31	−82.4 (5)
C11—C10—Fe1—C15	−78.3 (5)	C37—C36—Fe2—C31	157.2 (5)
C9—C10—Fe1—C15	162.6 (4)	C35—C36—Fe2—C33	−165.3 (4)
C11—C10—Fe1—C13	−160.5 (4)	C37—C36—Fe2—C33	74.3 (6)
C9—C10—Fe1—C13	80.3 (5)	C35—C36—Fe2—C34	37.8 (4)
C11—C10—Fe1—C14	−119.6 (4)	C37—C36—Fe2—C34	−82.5 (5)
C9—C10—Fe1—C14	121.3 (4)	C37—C36—Fe2—C35	−120.4 (7)
C11—C10—Fe1—C12	172.0 (7)	C35—C36—Fe2—C30	−48.4 (9)
C9—C10—Fe1—C12	52.9 (9)	C37—C36—Fe2—C30	−168.8 (6)
C11—C10—Fe1—C9	119.1 (6)	C35—C36—Fe2—C38	81.8 (5)
C11—C10—Fe1—C7	37.3 (4)	C37—C36—Fe2—C38	−38.6 (5)
C9—C10—Fe1—C7	−81.9 (4)	C35—C36—Fe2—C37	120.4 (7)
C11—C10—Fe1—C8	81.4 (4)	C35—C36—Fe2—C29	161.6 (8)
C9—C10—Fe1—C8	−37.8 (4)	C37—C36—Fe2—C29	41.2 (12)
C11—C10—Fe1—C16	−50.3 (8)	C35—C36—Fe2—C32	−124.3 (4)
C9—C10—Fe1—C16	−169.4 (5)	C37—C36—Fe2—C32	115.4 (5)
C8—C9—Fe1—C11	−81.1 (4)	C5—C4—N1—N2	0.5 (7)
C10—C9—Fe1—C11	37.8 (4)	C3—C4—N1—N2	179.6 (5)
C8—C9—Fe1—C15	−160.3 (6)	C5—C4—N1—C2	−178.8 (6)
C10—C9—Fe1—C15	−41.4 (8)	C3—C4—N1—C2	0.3 (10)
C8—C9—Fe1—C13	122.6 (4)	O1—C2—N1—C4	5.4 (11)
C10—C9—Fe1—C13	−118.6 (4)	C1—C2—N1—C4	−173.7 (6)
C8—C9—Fe1—C14	165.5 (4)	O1—C2—N1—N2	−173.9 (6)
C10—C9—Fe1—C14	−75.7 (5)	C1—C2—N1—N2	7.0 (8)
C8—C9—Fe1—C12	81.0 (4)	C5—C6—N2—N1	1.0 (6)
C10—C9—Fe1—C12	−160.1 (4)	C7—C6—N2—N1	−179.1 (5)
C8—C9—Fe1—C10	−118.9 (6)	C4—N1—N2—C6	−0.9 (6)
C8—C9—Fe1—C7	−37.6 (4)	C2—N1—N2—C6	178.4 (5)
C10—C9—Fe1—C7	81.2 (4)	C18—C19—N3—N4	−0.4 (7)
C10—C9—Fe1—C8	118.9 (6)	C20—C19—N3—N4	178.3 (5)
C8—C9—Fe1—C16	48.0 (9)	C18—C19—N3—C22	−175.7 (6)
C10—C9—Fe1—C16	166.9 (7)	C20—C19—N3—C22	3.1 (10)
C8—C7—Fe1—C11	118.8 (5)	O2—C22—N3—C19	−1.6 (10)
C6—C7—Fe1—C11	−121.3 (7)	C21—C22—N3—C19	178.2 (6)
C11—C7—Fe1—C15	75.8 (4)	O2—C22—N3—N4	−176.7 (6)
C8—C7—Fe1—C15	−165.4 (4)	C21—C22—N3—N4	3.1 (9)
C6—C7—Fe1—C15	−45.5 (6)	C18—C17—N4—N3	−0.8 (7)
C11—C7—Fe1—C13	−164.7 (7)	C16—C17—N4—N3	−177.1 (5)
C8—C7—Fe1—C13	−45.9 (9)	C19—N3—N4—C17	0.8 (7)
C6—C7—Fe1—C13	74.0 (10)	C22—N3—N4—C17	176.6 (5)
C11—C7—Fe1—C14	39.5 (8)	C27—C26—N5—N6	0.5 (7)
C8—C7—Fe1—C14	158.2 (6)	C25—C26—N5—N6	−179.2 (5)
C6—C7—Fe1—C14	−81.9 (9)	C27—C26—N5—C24	175.6 (6)
C11—C7—Fe1—C12	160.0 (4)	C25—C26—N5—C24	−4.1 (10)
C8—C7—Fe1—C12	−81.2 (4)	O3—C24—N5—N6	−178.9 (5)
C6—C7—Fe1—C12	38.7 (7)	C23—C24—N5—N6	0.5 (8)
C11—C7—Fe1—C10	−37.6 (4)	O3—C24—N5—C26	6.3 (10)
C8—C7—Fe1—C10	81.2 (4)	C23—C24—N5—C26	−174.3 (6)
C6—C7—Fe1—C10	−158.9 (6)	C27—C28—N6—N5	0.2 (6)
C11—C7—Fe1—C9	−81.4 (4)	C29—C28—N6—N5	179.4 (5)
C8—C7—Fe1—C9	37.3 (3)	C26—N5—N6—C28	−0.4 (6)
C6—C7—Fe1—C9	157.2 (6)	C24—N5—N6—C28	−176.1 (5)
C11—C7—Fe1—C8	−118.8 (5)	C40—C41—N7—N8	−1.4 (7)
C6—C7—Fe1—C8	119.9 (7)	C42—C41—N7—N8	177.7 (6)
C11—C7—Fe1—C16	118.0 (4)	C40—C41—N7—C44	177.7 (6)
C8—C7—Fe1—C16	−123.2 (4)	C42—C41—N7—C44	−3.2 (10)
C6—C7—Fe1—C16	−3.3 (6)	O4—C44—N7—C41	−3.6 (11)
C9—C8—Fe1—C11	81.9 (4)	C43—C44—N7—C41	178.0 (6)
C7—C8—Fe1—C11	−37.7 (3)	O4—C44—N7—N8	175.4 (6)
C9—C8—Fe1—C15	157.7 (7)	C43—C44—N7—N8	−3.0 (9)
C7—C8—Fe1—C15	38.1 (9)	C40—C39—N8—N7	0.2 (7)
C9—C8—Fe1—C13	−76.9 (4)	C38—C39—N8—N7	−178.7 (5)
C7—C8—Fe1—C13	163.5 (3)	C41—N7—N8—C39	0.7 (7)
C9—C8—Fe1—C14	−36.6 (9)	C44—N7—N8—C39	−178.5 (5)

### Hydrogen-bond geometry (Å, °)

**Table:**

*D*—H···*A*	*D*—H	H···*A*	*D*···*A*	*D*—H···*A*
C1—H1A···O2^i^	0.96	2.60	3.554 (8)	175
C10—H10···O1^ii^	0.93	2.57	3.490 (8)	170
C14—H14···O2^ii^	0.93	2.57	3.468 (8)	164
C43—H43A···O3^iii^	0.96	2.46	3.383 (9)	161
C31—H31···O3^ii^	0.93	2.59	3.515 (7)	172
C36—H36···O4^ii^	0.93	2.44	3.326 (8)	159

Symmetry codes: (i) −*x*+2, −*y*+2, −*z*+1; (ii) *x*−1, *y*, *z*; (iii) −*x*+5/2, *y*+1/2, −*z*+3/2.

## References

[bb1] Burla, M. C., Caliandro, R., Camalli, M., Carrozzini, B., Cascarano, G. L., De Caro, L., Giacovazzo, C., Polidori, G. & Spagna, R. (2005). *J. Appl. Cryst.* **38**, 381–388.

[bb2] Chakrabarty, S., Poddar, R. K., Poulsen, R. D., Thompson, A. L. & Howard, J. A. K. (2004). *Acta Cryst.* C**60**, m628–m630. 10.1107/S0108270104026459 15579948

[bb3] Enraf–Nonius (1989). *CAD-4 Software* Enraf–Nonius, Delft, The Netherlands.

[bb4] Esquius, G., Pons, J., Yáñez, R. & Ros, J. (2001). *J. Organomet. Chem.* **619**, 14–23.

[bb5] Farrugia, L. J. (1999). *J. Appl. Cryst.* **32**, 837–838.

[bb6] Harms, K. & Wocadlo, S. (1995). *XCAD4* University of Marburg, Germany.

[bb7] Miranda, C., Escartí, F., Lamarque, L., García-España, E., Navarro, P., Latorre, J., Lloret, F., Jiménez, H. R. & Yunta, M. J. R. (2005). *Eur. J. Inorg. Chem.* pp. 189–208.

[bb8] North, A. C. T., Phillips, D. C. & Mathews, F. S. (1968). *Acta Cryst.* A**24**, 351–359.

[bb9] Sheldrick, G. M. (2008). *Acta Cryst.* A**64**, 112–122. 10.1107/S0108767307043930 18156677

[bb10] Shi, Y.-C., Sui, C.-X. & Cheng, H.-J. (2005). *Acta Cryst.* E**61**, m1563–m1565.

[bb11] Shi, Y.-C., Zhu, B.-B. & Sui, C.-X. (2006*a*). *Acta Cryst.* E**62**, m2389–m2391.

[bb12] Shi, Y.-C., Zhu, B.-B. & Sui, C.-X. (2006*b*). *Acta Cryst.* E**62**, m2461–m2463.

[bb13] Spek, A. L. (2009). *Acta Cryst.* D**65**, 148–155. 10.1107/S090744490804362X PMC2631630 19171970

[bb14] Westrip, S. P. (2010). *J. Appl. Cryst.* **43**, 920–925.

